# Supplementary material for: Risk factors for sacrococcygeal pilonidal sinus: a systematic review and meta-analysis supplemented by genetic causal assessment
Source: Front Surg. 2026 Jan 7;12:1718589. doi: 10.3389/fsurg.2025.1718589 (PMC12819706; doi:10.3389/fsurg.2025.1718589)
Supplement: Supplementary file 2 [file Datasheet2.zip › Supplementary Data 2/MR_pipeline_p5e-8/forest_plots/finngen_R12_M13_ANKYLOSPON_STRICT.finngen_R12_L12_PILONIDALCYST.pdf]

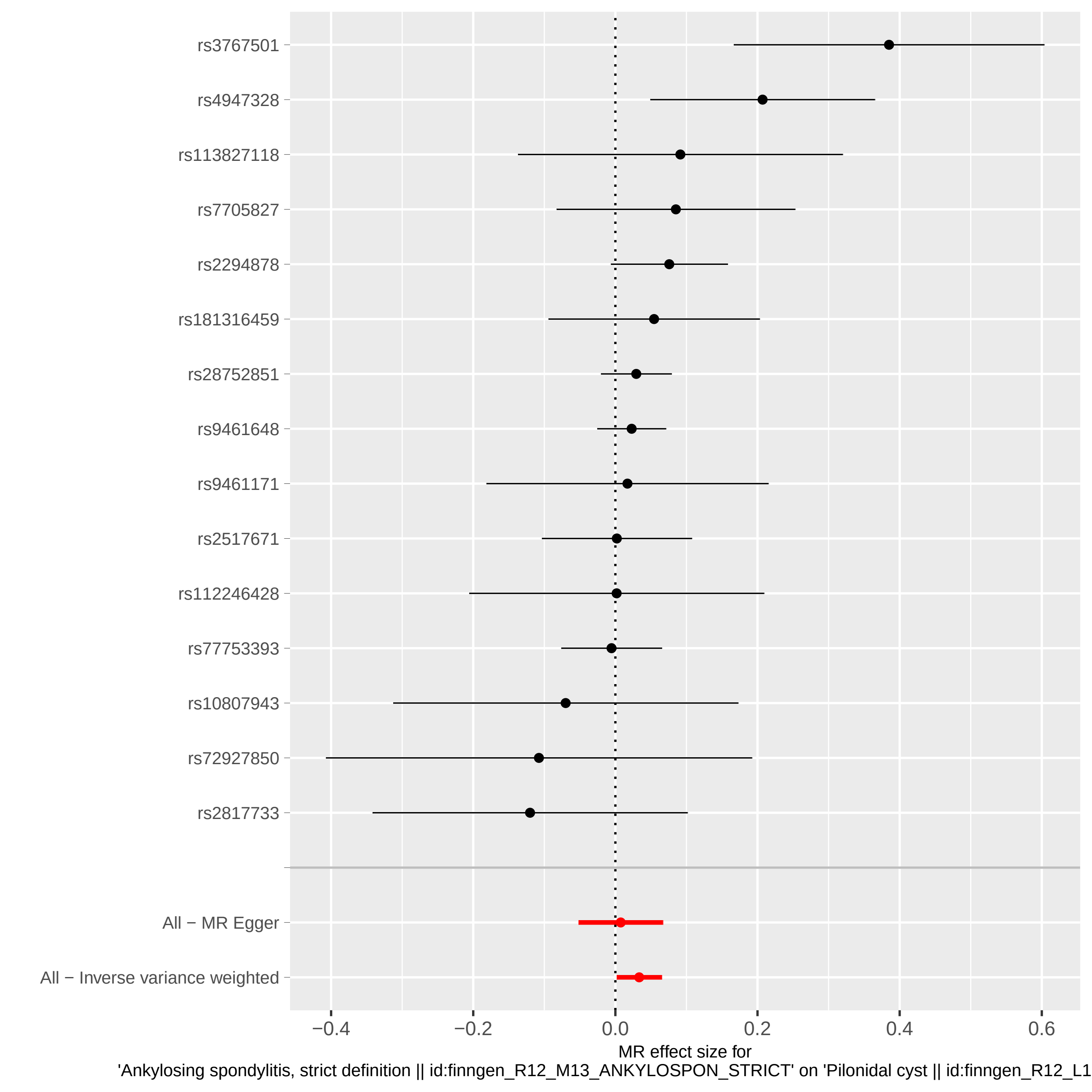

rs3767501
rs4947328
rs113827118
rs7705827
rs2294878
rs181316459
rs28752851
rs9461648
rs9461171
rs2517671
rs112246428
rs77753393
rs10807943
rs72927850
rs2817733
All − MR Egger
All − Inverse variance weighted
−0.4
−0.2
0.0
0.2
0.4
0.6
MR effect size for
'Ankylosing spondylitis, strict definition || id:finngen_R12_M13_ANKYLOSPON_STRICT' on 'Pilonidal cyst || id:finngen_R12_L1
